# Early-life Determinants of Stunted Adolescent Girls and Boys in Matlab, Bangladesh

**Published:** 2008-06

**Authors:** Alinda M. Bosch, Abdullah H. Baqui, Jeroen K. van Ginneken

**Affiliations:** ^1^Department of Population and Development, Netherlands Interdisciplinary Demographic Institute, The Hague, The Netherlands; ^2^Department of International Health, Johns Hopkins Bloomberg School of Public Health, 615 North Wolfe Street, Room E8138, Baltimore, MD, USA; ^3^ICDDR,B, GPO Box 128, Dhaka 1000, Bangladesh

**Keywords:** Adolescence, Anthropometry, Infant growth, Longitudinal studies, Maternal height, Nutritional status, Stunting, Bangladesh

## Abstract

This paper presents the results of a longitudinal study, conducted in Matlab, Bangladesh, that examined to what extent the level of stunting in adolescence can be predicted by nutritional status in early childhood and maternal height. A linked set of data collected from the same individuals at two moments in time, i.e. early childhood (1988–1989) and adolescence (2001), was analyzed. The study found that the odds of being stunted in adolescence could be explained by the combined effect of being stunted in childhood and having a mother whose height was less than 145 cm. Also, girls were more likely than boys to be stunted in childhood, whereas boys were more likely than girls to be stunted in adolescence. The latter is probably attributable to differences in the pace of maturation. In terms of policy and (reproductive health) programmes, it is important to recall that adolescent girls whose height and weight were subnormal (weight <45 kg and height <145 cm) might run an obstetric risk. Following these cut-off points, 83% and 23% of 16-year-old girls in this study would face obstetric risk, respectively, for weight and height if they marry and become pregnant soon.

## INTRODUCTION

Heredity determines how tall one can grow, whereas how tall one actually becomes depends partly on nutrition (1). The stature of a mother is probably the best predictor of the height of a child (2-3). The influence from the paternal side seems far less significant (2). Following recommendations of the United Nations Sub-Committee on Nutrition (4), nutritional status should be studied from a life-course (life-cycle) perspective. The (inter-related) effects of early-life growth failure may be passed on from one stage in life to the next via the mechanisms of ‘programming' as proposed by Barker (5) and ‘cumulative nutritional deprivation'. An inadequate dietary intake in the first years of life results in weight loss, growth faltering, lowered immunity, and mucosal damage, which, in turn, elevates the risks of infectious diseases. In addition, the metabolic system may be further altered. Early-life growth failure could consequently be passed on to the next generation, resulting in an intergenerational cycle of growth failure. This cycle starts before birth. Low pre-pregnancy nutritional status and insufficient weight gain of the mother during pregnancy are believed to be the main causes of low-birthweight babies (6-7). However, evidence of the relative contribution of genetic inheritance and intrauterine factors on weight and size at birth is inconclusive (8-15), as is the study on its relation to early-life anthropometry and health later in life (5,16-18).

Regarding long-term effects, Cole concluded that the increment in adult height is set by the age of two years, and he suggests that growth at this time in early life is the outcome of an interaction between concurrent nutrition and the growth rate set during pregnancy, reflecting parental size (19). Accordingly, it is believed that, although some height differences between people are attributed to gene-tics, the general trend for average height to increase is almost certainly due to improvements in nutrition and, to a lesser extent, health (20). Chronic malnutrition and disease in childhood may stunt growth, and potential adult height may not be reached (21). Particularly when children remain in poor environments, the potential for catch-up faltering growth in childhood is believed to be limited after the age of two years (7). There is little evidence that growth retardation suffered in early childhood can be significantly compensated for in adolescence (22). There is, however, evidence, although based on a small-scale (n=60) study, that chronic undernutrition in girls retards skeletal growth and maturation and postpones menarche. However, it does not have an impact on the magnitude of the (adolescent) height spurt, and it also does not result in a lower adult stature because of an extended length of the growth period (23). Also, results of adoption studies showed that some catch-up might be possible due to an extended growing period, but this is not complete for children who remain in an adverse environment (as in Bangladesh), and the effect may be more pronounced for boys than for girls (24-25). Within the context of foetal programming, boys are in general more sensitive to nutritional deprivation than girls (16). Catch-up growth that takes the form of an accelerated growth may also trigger early puberty which limits final height (26).

Within Bangladeshi society, however, during infancy and childhood, the opposite—girls being more vulnerable than boys—may be true as a result of an unequal allocation of food and care at the expense of girls. Although girls survive in greater numbers than infant boys, almost everywhere, in a few countries including Bangladesh, gender discrimination outweighs the biological advantage of girls (22,27-29). Ramifications may be gender differentials in nutritional status and mortality from illness (27,30-33). There are indications that discrimination against girls in Bangladesh is negligible in small families but much more pronounced in families with more than two girls (34). In Bangladesh, malnutrition among children and women seems persistent (6,35-37): 50% of babies have low birthweight, 48% and 45% of children aged less than five years (under-five children) are, respectively, underweight and stunted, 73% of under-five children and 74% of pregnant women are anaemic, and 47% of women have a body mass index (BMI) below 18.5, indicating underweight (22).

In this study, it is hypothesized that stunted adolescents are more likely to have stunted mothers and that they are more likely to have been stunted in early childhood.

## MATERIALS AND METHODS

The study was conducted in three villages in Matlab, a rural area of Bangladesh. ICDDR,B has been maintaining a Health and Demographic Surveillance System (HDSS) in Matlab since 1966. Details of the study villages and selection criteria have been reported elsewhere (38).

The study involved the follow-up of 707 under-five children (387 boys and 320 girls) who were enrolled in an earlier study on childhood infectious diseases, conducted in Matlab during 1988–1989, by the second author of this paper. Findings and details of this baseline study have been described elsewhere (39-42). At the start of the baseline study, in April 1988, the youngest child enrolled was aged less than one month whereas the oldest child was aged almost five years. The follow-up study took place in 2001. By that year, under-five children had grown up to be adolescents aged 12–16 years. Linking of individual records was feasible because of the Registration Identification (RID) number used in the HDSS. In total, 562 adolescents (307 boys and 255 girls) were eligible for follow-up. Analyses of lost cases due to migration and death have been described elsewhere (38). At baseline, cases lost to follow-up did not differ significantly from those who were successfully followed up in terms of nutritional status and selected sociodemographic conditions. Informed written consent was obtained from the mother of each participating under-five child before enrollment in the baseline study and from adolescents and also from their mothers or fathers in the follow-up study. Both at baseline and at follow-up, the HDSS provided the date of birth. The HDSS data are highly accurate. Chronological age was assessed based on the date of birth and date of interview.

At baseline and at follow-up, trained research assistants and interviewers took anthropometric measurements. At baseline, each under-five child was weighed nude or with light clothes to the nearest 0.1 kg with a Salter-type spring scale. At follow-up, each adolescent was weighed wearing daily clothes to the nearest 0.1 kg with a digital-weighing scale, following the guidelines of the World Health Organization (WHO) (43). In both the surveys, the scales were calibrated against known weights before use. Recumbent length of children aged less than 36 months and standing height of children aged ≥36 months and of adolescents and their mothers were measured to the nearest 0.1 cm using a locally-constructed length board. To reduce observation errors, two observers read anthropometric measurements independently, both at baseline and at follow-up, and weight was measured twice. The mean of the two measurements was taken as the actual value. At baseline, 10% of the children were measured again the following day to assure the quality of the weight and height measurements. At follow-up, 10% of the adolescents and their mothers were measured again.

In the linked dataset, valid anthropometric data were available for 482 under-five children and 485 adolescents respectively. Their nutritional status was assessed based on the international anthropometric indices recommended by the Centers for Disease Control and Prevention (CDC) and WHO: weight-for-age or undernutrition, and height-for-age or stunting, and—only in adolescence—body mass index (BMI), indicating ‘thinness' or chronic energy deficiency (CED) (22,43). Data on nutritional status were analyzed using the nutritional anthropometry program ‘NutStat' of EPI Info (version 1.1.2) (44). Two different reference populations of the United States National Center for Health Statistics (NCHS) were applied: the CDC/WHO reference population of 1978 to the nutritional analyses of under-five children in 1988–1989 and the CDC reference population of 2000 to the nutritional analyses of the same population in adolescence in 2001. The rationale for this selection was that these two reference populations were as close as possible in time to the year of measurement of anthropometry of the study population in childhood and adolescence respectively. In the baseline study, the under-five children were measured a variable number of times, with a maximum of 14, within an approximate two-year period. For the follow-up study, early childhood nutritional status was determined by an average of up to 14 consecutive anthropometric indices.

The level of stunting in adolescence—the dependent variable—was expressed as a dichotomous variable, i.e. category 0 (not stunted or >-2 SD) and category 1 (stunted or ≤−2 SD). The level of underweight in early childhood and the level of underweight in adolescence were expressed as an ordinal variable. Category 1 (not underweight) is >−2 SD from the median of the reference population; category 2 (moderately underweight) is between −3 SD and −2 SD from the median of the reference population; and category 3 (severely underweight) is <−3 SD from the median of the reference population. The cut-off points for the three levels of childhood stunting, i.e. not stunted, moderately stunted, and severely stunted, were the same as those of underweight, as were the cut-off points for the three levels of adolescent CED, expressed by BMI z-scores, i.e. not CED, moderately CED, severely CED.

Maternal height was also dichotomous, including category 0 (<145 cm) and category 1 (≥145 cm), following the cut-off point for height below which there might be an obstetric risk (22). In the linked dataset, data on height were available for 436 mothers. Finally, sex was coded as 0 for female and 1 for male.

Kendall's *tau-b* coefficient, a measure of association for nominal data, was used for indicating the strengths and directions of correlation of adolescent stunting with potential predictors. Also, the extent of multi-collinearity between the independent variables was assessed. By applying binary logistic regression models (with 95% confidence interval [CI]), it was aimed to build up a ‘stunting profile' to determine which adolescents, given a series of indicators pertaining to nutritional status earlier in life, are most likely to be stunted. First, the effects of single independent variables or covariates (bivariate model) were studied, and in a second step, independent variables were controlled for (multivariate analyses). In the models, the independent variables or covariates were categorical. The categories were compared with the reference category (ref.), whereby the latter encompass boys and girls who were respectively not malnourished in childhood according to anthropometry, or who had a mother who was 145 cm or taller. In the tables, only the main parameter of the model (B) and the level of significance are shown, along with the corresponding standard error (SE). Nagelkerke's R^2^ indicates the percentage of variance in the dependent variable (stunting in adolescence) explained by the predictors (independent variables) included in the model.

In model 1, the effects of childhood underweight and childhood stunting on stunting status in adolescence were examined. In model 2, the effect of maternal height was added whereas in model 3, the effect of sex of the child was explored. In model 4, both effect of maternal height and effect of sex were taken into account but left out the variable—underweight in childhood—because of its insignificant contribution. In the final model—model 5—, the effects of all potential predictors of stunting status in adolescence were explored all together.

## RESULTS

Table 1 presents the main overall and nutritional characteristics of the study population in rural Matlab in early childhood and adolescence and height of the mother. The population appeared to be largely malnourished: 66% of the adolescent boys and 46% of the adolescent girls were severely underweight whereas 36% and 28% of the adolescent boys and girls were respectively severely stunted.

**Table 1:** Characteristics of study population in early childhood and adolescence

Characteristics	Boys	Girls	Total
Under-five children at baseline, 1988–1989 (38-42)	387		
Adolescents at follow-up, 2001, no. (%)	307 (79)		
Age composition (years) in adolescence (%)			
12	12	16	14
13	26	16	22
14	26	36	30
15	26	18	22
16	10	15	12
Average weight (kg) in adolescence by age (years)			
12	25.6	27.6	26.6
13	27.4	29.1	28.0
14	31.8	33.4	32.7
15	35.1	36.9	35.8
16	38.7	39.1	39.0
Average height (cm) in adolescence by age (years)			
12	132.9	136.5	134.7
13	137.0	138.5	137.5
14	143.6	144.3	144.0
15	148.8	146.6	148.0
16	153.5	149.2	151.1
Severe underweight (below −3 SD from median) (%)			
In early childhood (n=482)*	23	36	29
In adolescence (n=485)^†^	66	46	57
Severe stunted (below −3 SD from median) (%)			
In early childhood (n=482)*	22	40	36
In adolescence (n-485)^†^	36	28	33
Severe chronic energy deficient in adolescence			
(BMI z-scores below −3 SD from median) by age (years) (%)			
12	22	17	19
13	25	15	22
14	25	11	20
15	30	17	23
16	30	15	23
Height of mother of child in adolescence (%)			
<145 cm (n=77)	15	20	18
≥145 cm (n=362)	85	80	82

*Using the CDC/WHO reference population of 1978 (US NCHS)

^†^Using the CDC reference population of 2000 (US NCHS); BMI=Body mass index; CDC=Centers for Disease Control and Prevention; NCHS=National Center for Health Statistics; SD=Standard deviation; WHO=World Health Organization

At every age, on average, the adolescent girls were heavier than boys. The adolescent girls also took the lead with regard to height, but this did not last for long: soon after reaching the age of 14 years, the adolescent boys caught up on their relative backlog. At the age of 15 years, the adolescent boys were, on average, 148.8 cm tall, whereas the mean height of the adolescent girls was 146.6 cm. The proportion of adolescents who had a mother with a low height was higher among girls (20%) than among boys (15%).

Evidence was found in cross-tabulations that the stunting status in adolescence was predisposed by nutritional status in early childhood. Of boys who were severely stunted as under-five, 71% remained severely stunted in adolescence (not shown). Also, 48% and 17% of not-stunted under-five boys, respectively, became moderately and severely stunted in adolescence. However, 54% of the girls who were not stunted as under-five remained not stunted as adolescent.

Table 2 shows the strengths and directions of correlation between the dependent variable (adolescent stunting, i.e. stunted versus not stunted) and the selected independent variables or predictors.

**Table 2:** Strengths and directions of correlation among selected variables, Matlab, Bangladesh, 1988-2001

Factor	Adolescent				Childhood		Maternal height	Sex
	Weight	Height	Underweight	BMI	Underweight	Stunting		
Adolescent stunting	−0.391**	−0.487**	0.555**	0.197**	0.191**	0.294**	−0.152**	0.118**
N	485	485	485	485	482	482	439	485
Adolescent weight		0.692**	-0.553**	−0.449**	−0.047	0.015	0.102**	−0.115**
N		485	485	485	482	482	439	485
Adolescent height			−0.429**	−0.177**	−0.028	−0.055	0.138**	−0.036
N			485	485	482	482	439	485
Adolescent underweight				0.566**	0.269**	0.249**	−0.112*	0.213**
N				485	482	482	439	485
Adolescent BMI z-scores					0.204**	0.083*	−0.033	0.237**
N					482	482	439	485
Childhood underweight						0.636**	−0.149**	−0.067
N						482	436	482
Childhood stunting							−0.171**	0.098*
N							436	482
Maternal height								−0.063
N								436

*Statistically significant at 0.05 level;

**Significant at ≥0.01 level; BMI=Body mass index

Adolescent stunting (highly) significantly correlated with all other nutritional indicators pertaining to the adolescent period, i.e. adolescent weight, height, underweight, and CED according to BMI z-scores. The significant correlation of adolescent stunting with adolescent height was negative, meaning that the lower an adolescent's height was the more likely he or she was stunted, i.e. short for his or her age compared to a well-nourished reference population. The significant correlation of adolescent stunting with adolescent underweight and CED according to BMI z-scores was positive, meaning that the less adequate an adolescent's nutritional status was according to these two indicators, the more likely that he or she was stunted.

Adolescent stunting furthermore (highly) significantly correlated with early childhood underweight and stunting (positive), meaning that the less adequate an adolescent's nutritional status was in early childhood according to the level of underweight and stunting at that time, the more likely it was that he or she was stunted in adolescence. Adolescent stunting (highly) significantly correlated with maternal height (negative), meaning that short mothers, i.e. mothers whose height was below 145 cm, were more likely to have an adolescent son or daughter who was stunted. Adolescent stunting (highly) significantly correlated with the sex of the adolescent (positive), meaning that a boy was more likely than a girl to be stunted in adolescence.

Some independent variables strongly correlated with each other (multi-collinearity). Collinearity between some variables was expected. For example, adolescent weight and height, for instance, (highly) significantly correlated with each other (positive), meaning that an adolescent who weighed more was also taller and vice-versa. Collinearity might also be due to the fact that measurements were based on the same anthropometric indices. For instance, adolescent underweight and adolescent CED according to BMI z-scores were both, in part, based on adolescent weight and, hence, also (highly) significantly correlated with each other (positive). However, collinearity was also present between indicators of nutritional status pertaining to different stages in life (not shown).

Given that adolescent stunting, the dependent variable, significantly correlated to most predictors included in the model, the regression models were reviewed to examine the effect of one predictor while controlling for possible confounders. In Table 3, the odds ratios (generated by the univariate model) are shown for the likelihood of being stunted in adolescence, respectively, by level of childhood underweight and stunting, maternal height and sex of the adolescent. The odds ratios were also estimated for boys and girls separately. Although adolescent stunting highly correlated with adolescent underweight and BMI, these two indicators could not have influenced the latter, since the anthropometric values on which they were based were taken at the same time. Hence, adolescent underweight and BMI were excluded from the regression models.

**Table 3:** Binary logistic bivariate regression models: odds ratios (with 95% CI) of adolescent stunting (total and by sex), Matlab, Bangladesh, 1988–2001

Factor	Odds ratio	Constant	Nagelkerke R^2^
Childhood underweight (weight-for-age)			
Total			
Not underweight: ≥2 SD (ref)	1.00	1.685**	0.060
Moderately underweight: −3 to −2 SD	1.75* (1.10–2.78)		
Severely underweight: ≤3 SD	3.56*** (1.99–6.36		
Boys			
Not underweight: ≥2 SD (ref)	1.00	2.800***	0.022
Moderately underweight: −3 to −2 SD	1.39 (0.71–2.72)		
Severely underweight: ≤3 SD	2.32 (0.94–5.73)		
Girls			
Not underweight: ≥2 SD (ref)	1.00	1.029	0.126
Moderately underweight: −3 to −2 SD	1.94 (0.99–3.81)		
Severely underweight: ≤3 SD	5.51*** (2.54–11.94)		
Childhood stunting (height-for-age)			
Total			
Not stunted: ≥2 SD (ref)	1.00	1.319	0.142
Moderately stunted: −3 to −2 SD	2.64*** (1.65–4.23)		
Severely stunted: ≤3 SD	7.40*** (3.87–14.15)		
Boys			
Not stunted: ≥2 SD (ref)	1.00	1.906**	0.127
Moderately stunted: −3 to −2 SD	2.59** (1.34–5.03)		
Severely stunted: ≤3 SD	9.61*** (2.79–33.18)		
Girls			
Not stunted: ≥2 SD (ref)	1.00	0.850	0.191
Moderately stunted: −3 to −2 SD	2.63** (1.32–5.24)		
Severely stunted: ≤3 SD	8.47*** (3.79–18.93)		
Maternal height			
Total			
145 cm or taller (ref)	1.00	2.415***	0.038
Shorter than 145 cm	3.13** (1.51–6.50)		
Boys			
145 cm or taller (ref)	1.00	3.326***	0.019
Shorter than 145 cm	2.41 (0.81-7.16)		
Girls			
145 cm or taller (ref)	1.00	1.717***	0.070
Shorter than 145 cm	4.19** (1.56–11.27)		
Sex of child			
Total			
Male (ref)	1.00	3.727***	0.020
Female	0.58** (0.39–0.88)		

*Statistically significant at 0.05 level

**Significant at 0.01 level

***Significant at 0.001 level and higher; CI=Confidence interval; Ref=Reference; SD=Standard deviation

Table 3 shows that there was a significant effect of underweight in childhood on stunting status in adolescence. The odds of being stunted in adolescence for children who were moderately underweight in childhood was 1.75 times the odds for children who had a normal weight according to their age and sex in childhood (reference category). In addition, the odds ratio was considerably higher for adolescents who were severely underweight in childhood (3.56). When the data were broken down by sex, the results lost significance for both boys and girls who were moderately underweight in early childhood whereas they remained significant for severely-underweight girls. This indicates that, for girls, childhood underweight was a better predictor of adolescent stunting than it was for boys. What really stood out was the highly significant (p<0.001) effect on the odds of being stunted in adolescence for children who were moderately and severely stunted in childhood compared to children who had a normal height according to their age and sex, i.e. not stunted under-five children. This effect remained significant when the data were broken down by sex. The odds of being stunted in adolescence (irrespective of sex) for children who were moderately stunted in childhood was 1.64 times the odds for children who were not stunted in childhood, whereas the odds of being stunted in adolescence for children who were severely stunted in childhood was even 7.40 times the odds for children who were not stunted in childhood (reference category). This effect was even stronger when the data were analyzed for the two sexes separately.

With regard to the effect of maternal height on the odds of being stunted in adolescence, also a highly significant figure could be found. However, this effect was again no longer significant when the data were analyzed for the two sexes separately. The odds of being stunted in adolescence for children (boys and girls considered together) who had a mother of short stature (<145 cm) was as high as 3.13 times the odds for children who had a taller mother (≥145 cm) (reference category).

Finally, the sex of the adolescent appeared to be a highly significant predictor of stunting status in adolescence. The odds of being stunted in adolescence for girls was 0.58 times the odds for boys (the reference category), meaning that girls were less likely than boys to be stunted in adolescence. The highest Nagelkerke R^2^ found was the one pertaining to childhood stunting: 0.142 for all children (and 0.191 for girls). This figure means that 14.2% of the variation in stunting in adolescence is explained by childhood stunting only. For girls, this variable explains 19.1% of the variation in adolescent stunting.

Finally, the most salient multivariate models are shown (Table 4).

**Table 4:** Binary logistic multivariate regression models: odds ratios (with 95% CI) of adolescent stunting controlled for selected variables, Matlab, Bangladesh, 1988–2001

Factor	Odds ratio				
	Model 1	Model 2	Model 3	Model 4	Model 5
Childhood underweight					
Not underweight: ≥2 SD (ref)	1.00	1.00	1.00	-	1.00
Moderately underweight: −3 to −2 SD	0.83 (0.47–1.47)	0.87 (0.48–1.58)	0.75 (0.42–1.34)		0.77 (0.42–1.43)
Severely underweight: ≤3 SD	0.88 (0.40–1.92	0.80 (0.36–1.80)	0.85 (0.38–1.88)		0.78 (0.34–1.77)
Childhood stunting					
Not stunted: ≥2 SD (ref)	1.00	1.00	1.00	1.00	1.00
Moderately stunted: −3 to −2 SD	2.90*** (1.64-5.14	2.83** (1.56–5.11)	3.01*** (1.68–5.40)	2.58*** (1.55–4.27)	2.96*** (1.61–5.42)
Severely stunted: ≤3 SD	8.10*** (3.55–18.45)	8.64*** (3.60–20.75)	9.90*** (4.23–23.17)	8.96*** (4.35–18.46)	10.53*** (4.29–26.35)
Maternal height					
145 cm or taller (ref)	-	1.00	-	1.00	1.00
Shorter than 145 cm	-	2.33* (1.09–4.99)	-	2.31* (1.08–4.96)	2.32* (1.08–4.98)
Sex of child					
Male (ref)	-	-	1.00	1.00	1.00
Female	-	-	0.43*** (0.28–0.68)	0.42*** (0.26–0.67)	0.41*** (0.25–0.66)
N	482	436	482	436	436
Nagelkerke R^2^	0.14	0.17	0.18	0.20	0.21
-2 log likelihood	500.15	445.03	486.30	432.95	431.26
Constant (exp(B))	1.39	1.23	2.10	1.74	1.90

*p<0.05

**p<0.01

***p<0.001

CI=Confidence interval; Ref=Reference; SD=Standard deviation

Table 4 reveals that childhood stunting was the most important highly significant predictor of adolescent stunting. For instance, model 1 showed that the odds of being stunted in adolescence for children who were moderately stunted in childhood was 2.90 times the odds for children who were not stunted in childhood (reference category).

The odds ratio of being stunted in adolescence for children who were severely stunted in childhood was even 8.10 times the odds for children who were not stunted in childhood (reference category). The significant effect of childhood underweight on the odds of being stunted in adolescence, which was observed in Table 3, is lost when childhood underweight was considered together with childhood stunting into one model. From model 2, 4, and 5, it could be learned that maternal height was also a significant predictor of the odds of adolescent stunting. The effect of maternal height on adolescent stunting holds when the sex of the child was taken into account (model 4 and 5). In model 3 through 5, the variable indicating sex of the child had a modest effect: basically, the odds of being stunted in adolescence for girls was about 0.4 times the odds for boys (reference category), meaning that girls were less likely to be stunted in adolescence compared to boys. The Nagelkerke R^2^ increased from 0.143 in model 1 to 0.211 in model 5. The latter figure indicates that 21.1% of the variation in adolescent stunting was explained by the combined effect of the predictors included in this model, notably childhood stunting, height of the mother, and sex of the child. A strength of Table 4 is that all models showed a convincing, stable pattern. A limitation might be that some nutritional status indicators were highly correlated, notably when related to the same period in live, such as childhood underweight and childhood stunting (Table 2).

## DISCUSSION

This study examined the predisposition of the level of stunting in adolescence by the level of stunting and underweight in early childhood, maternal height, and sex of the child. The adolescent population in the sample can be considered to be largely malnourished. It was found that adolescent girls were less likely to be malnourished than boys. The binary logistic regression analyses revealed, for instance, that the odds of being stunted in adolescence for girls was about 0.4 times the odds for boys (reference category), meaning that girls were less likely to be stunted in adolescence than boys.

The differences between boys and girls may be related to the combined effect of the difference in timing of the adolescent growth spurt and the possible difference in catch-up potential within the context of malnutrition. As girls enter puberty two years earlier than boys, their growth ceases at least two years before that of boys, and these two years also represent the difference in the peak of height velocity between the sexes (45). In early childhood, however, girls are relatively more often severely underweight and severely stunted than boys. However, if the two categories (moderate and severe stunting) were considered together, this difference was almost counterbalanced: the percentage of boys and girls who were stunted in early childhood is then 64 against 67.

It was found that whereas, on average, adolescent girls were heavier compared to boys throughout the early and middle adolescent period, boys ultimately seemed to grow taller than girls. The turning point in height, i.e. when adolescent boys in the sample catch-up with their female counterparts, is right after the age of 14 years. Timing and tempo of changes in height, weight, and body composition in adolescence vary greatly by sex: lean body mass may attain its adult level as early as by the fourteenth year in girls, but the growth spurt usually subsides at the age of sixteen (21) whereas in boys, adult height is reached later, possibly as late as at the ages of 17–18 years (22,46). It was also studied to what extent low height might become an embedded trait and passed on from mother to child. The proportion of adolescents who had a mother with a low height was 18%. Particularly, a highly significant association between height of the mother and stunting status of the adolescent son or daughter was found.

The binary logistic regression analyses (bivariate models) revealed that, irrespective of sex, the odds of being stunted in adolescence for children who were moderately stunted in childhood was 1.64 times the odds for children who were not stunted in childhood whereas the odds of being stunted in adolescence for children who were severely stunted in childhood was 7.40 times the odds for children who were not stunted in childhood (reference cate-gory). When taking all potential nutritional indicators together into consideration, it appeared that variation in stunting in adolescence was explained by the combined effect of the predictors included in this model, notably childhood stunting, height of the mother, and sex of the child.

A limitation of the study was that the study population was not followed up through adulthood. Information about catch-up growth in later adolescence (by means of a prolonged growth period) was lacking, and, hence, no conclusions could be drawn on the ultimate effects of childhood undernutrition on adult stature. Having said this, it should be noted that, in terms of reproductive health, there are reasons for concern: the adolescent girls in our sample were likely not to have completed growth but their height and weight seemed also to be sub-normal because of malnutrition. In absolute terms, some girls in this study were at risk because their weight and height fell below the cut-off points below which obstetric risks increase. Specifically, if the 16-year-old girls in our sample married and become pregnant soon afterwards, 83% and 23% would be at risk in terms of obstetric cut-off points, respectively, for weight and height.

Programmes aimed at improving the nutritional status in adolescence should, therefore, explicitly take the period of early childhood and the period of gestation into account. A central contention of studies undertaken by Barker and advocates is that babies with thrifty phenotypes, i.e. babies who are ‘designed' to live in an environment that is chronically short on food and who subsequently grow up in affluent environments, ‘may operate suboptimally' (20). Given this notion, supplementing the diets of pregnant women whose children are likely to remain in a thrifty environment would be counterproductive. Thus, rather than improving nutritional conditions in general, a specific approach may be needed whereby the key message could be to harmonize prenatal (in utero) nutritional conditions—the ‘maternal nutritional forecast'—with the postnatal nutritional environment. Obviously, this recommendation should not be interpreted as an appeal to leave children in a poor nutritional environment (or to leave the poor as they are), but calls instead for a carefully-monitored nutritional intervention programme whereby food supply is guaranteed for a longer period than just the pregnancy itself.
